# Pathogenic mitochondrial DNA variants are associated with response to anti-VEGF therapy in ovarian cancer PDX models

**DOI:** 10.1186/s13046-024-03239-w

**Published:** 2024-12-19

**Authors:** Daniele Boso, Ilaria Piga, Chiara Trento, Sonia Minuzzo, Eleonora Angi, Luisa Iommarini, Elisabetta Lazzarini, Leonardo Caporali, Claudio Fiorini, Luigi D’Angelo, Monica De Luise, Ivana Kurelac, Matteo Fassan, Anna Maria Porcelli, Filippo Navaglia, Ilaria Billato, Giovanni Esposito, Giuseppe Gasparre, Chiara Romualdi, Stefano Indraccolo

**Affiliations:** 1 Basic and Translational Oncology Unit, Veneto Institute of Oncology IOV-IRCCS, Padua, Italy; 2 Department of Surgery, Oncology and Gastroenterology, University of Padova, Via Gattamelata, 64 - 35128 Padua, Italy; 3 Department of Pharmacy and Biotechnology (FABIT), University of Bologna, Bologna, Italy; 4 IRCCS Istituto Delle Scienze Neurologiche Di Bologna, Bologna, Italy; 5 Department of Medical and Surgical Sciences (DIMEC), University of Bologna, Bologna, Italy; 6 Center for Applied Biomedical Research (CRBA), University of Bologna, Bologna, Italy; 7 Department of Medicine (DIMED), University of Padua, Padua, Italy; 8 Veneto Institute of Oncology, IOV-IRCCS, Padua, Italy; 9 IRCCS Azienda Ospedaliero-Universitaria Di Bologna, Bologna, Italy; 10 Laboratory Medicine, Department of Medicine-DIMED, University Hospital of Padova, Padua, Italy; 11 Department of Biology, University of Padova, Padua, Italy; 12 Immunology and Molecular Oncology Unit, Istituto Oncologico Veneto, IOV - IRCCS, Padua, Italy; 13 Centro Studi E Ricerca Sulle Neoplasie Ginecologiche (CSR), University of Bologna, Bologna, Italy; 14 Department of Pharmacy and Biotechnology (FABIT) and Interdepartmental Center for Industrial Research On Health Sciences and Technologies, University of Bologna, Bologna, Italy

**Keywords:** Ovarian cancer, Mitochondrial DNA mutations, Patient-derived xenografts, Antiangiogenic therapy, Predictive biomarkers

## Abstract

**Background:**

Mitochondrial DNA (mtDNA) pathogenic variants have been reported in several solid tumors including ovarian cancer (OC), the most lethal gynecologic malignancy, and raised interest as they potentially induce mitochondrial dysfunction and rewiring of cellular metabolism. Despite advances in recent years, functional characterization of mtDNA variants in cancer and their possible modulation of drug response remain largely uncharted.

**Methods:**

Here, we characterized mtDNA variants in OC patient derived xenografts (PDX) and investigated their impact on cancer cells at multiple levels.

**Results:**

Genetic analysis revealed that mtDNA variants predicted as pathogenic, mainly involving complex I and IV genes, were present in all but one PDX (*n* = 20) at different levels of heteroplasmy, including 7 PDXs with homoplasmic variants. Functional analyses demonstrated that pathogenic mtDNA variants impacted on respiratory complexes activity and subunits abundance as well as on mitochondrial morphology. Moreover, PDX cells bearing homoplasmic mtDNA variants behaved as glucose-addicted and could barely survive glucose starvation in vitro. RNA-seq analysis indicated that mtDNA mutated (heteroplasmy > 50%) PDXs were endowed with upregulated glycolysis and other pathways connected with cancer metabolism. These findings led us to investigate whether pathogenic mtDNA variants correlated with response to anti-VEGF therapy, since the latter was shown to reduce glucose availability in tumors. Strikingly, PDXs bearing homoplasmic pathogenic mtDNA variants associated with improved survival upon anti-VEGF treatment in mice, compared with mtDNA wild type or low heteroplasmy PDXs.

**Conclusions:**

These results hint at mtDNA variants as potential biomarkers of response to antiangiogenic drugs.

**Supplementary Information:**

The online version contains supplementary material available at 10.1186/s13046-024-03239-w.

## Background

Epithelial ovarian cancer (EOC) represents the leading cause of death in gynecological malignancies [1]. Poor prognosis is often attributed to late diagnosis, acquired resistance to platinum-based regimen and recurrence [2]. Cytoreductive surgery and platinum/paclitaxel chemotherapy are the standard therapeutic approaches [1]. Furthermore, maintenance therapy with PARP inhibitors or antiangiogenic drugs is nowadays recommended in national guidelines for treatment of advanced ovarian cancer [3]. Growth of EOC cells is dependent on vascular endothelial growth factor (VEGF) mediated angiogenesis [4]. Bevacizumab is a humanized monoclonal antibody that, by binding to VEGFA, inhibits the activation of its receptor and thus reduces tumor neo-angiogenesis [5]. Bevacizumab is now approved for both upfront and recurrent disease (platinum-sensitive and -resistant) in combination with carboplatin and paclitaxel based on multiple randomized phase 3 trials [2]. The addition of bevacizumab to standard chemotherapy improved progression-free survival (PFS); however, results were inconsistent in terms of overall survival (OS), underscoring the need to identify biomarkers of response. Several studies have investigated the presence of a marker of bevacizumab efficacy, with limited or incomplete results. In the context of EOC, a recent phase III trial (Gynecologic Oncology Group, GOG-0218) demonstrated that bevacizumab benefit could be predicted by high CD31 expression within the tumor [6]. Furthermore, significant effort has been dedicated to serum-based biomarker research, and while the majority of studies have yielded negative results, there has been evidence of a correlation between serum IL-6 levels and the benefit derived from bevacizumab [7]. However, these biomarkers await independent validation before they can be used in clinical practice for patient stratification purposes.

In experimental tumor models, it is well known that anti-angiogenic therapy leads to reduction in microvessel density as well as to increased hypoxia [8]. We previously found that intra-tumoral concentrations of ATP and glucose are dramatically reduced by anti-VEGF therapy and that the tumor response to these metabolic changes was largely dependent on the metabolic set-up of the tumor [12]. Our findings suggested that highly glycolytic tumors could better respond to VEGF targeting drugs. Along this line, Pommier et al. subsequently reported that a metabolic gene expression signature, comprising genes involved in glucose metabolism, angiogenesis, hypoxia, glutamine metabolism, and leptin signaling, correlated with response in colorectal cancer patients treated with an anti-angiogenic tyrosine kinase inhibitor [13]. In general, however, the clinical relevance of tumor metabolic features as predictive biomarkers of response to anti-VEGF therapy remains largely undetermined.

Mitochondrial DNA (mtDNA) is a small, circular 16.5-kb genome, coding for 22 tRNAs, 2 rRNAs and 13 essential subunits of Complex I, III, and IV of the electron transport chain and Complex V/ATP synthase, which are critical to oxygen-dependent cellular metabolism [14, 15]. Mitochondria play a pivotal role in energy production, cellular homeostasis, and apoptosis. Mitochondrial dysfunction, due to alterations of genes encoding mitochondrial proteins or other factors, has been associated with upregulation of glucose metabolism in tumors, and is considered a prominent hallmark of cancer pathogenesis. Previous investigations have elucidated the prevalence and distribution of mtDNA variants in various cancer types [14]. Notably, many tumors (~ 50%) are endowed with detectable somatic variants in the mtDNA genome, with high frequency in colorectal, thyroid and some types of renal cancer [15]. The vast majority of the described somatic mtDNA variants in cancer are heteroplasmic, reaching allele frequencies far below 100%, consequently most mtDNA-mutant tumors retain mitochondrial respiratory capacity in the form of wild-type mtDNA [15]. However, pathogenic mtDNA variants at high level of heteroplasmy likely influence cellular metabolism, through an impaired ATP production and a compensatory shift towards glycolysis, known as the Warburg effect, eventually contributing to tumorigenesis and cancer progression in dependence of the mutation type and load [13–15, 13, 14]. Although several studies described the presence of mtDNA mutations in ovarian cancer [15, 13], their functional characterization is still largely lacking as their potential impact on response to anti-angiogenic drugs. In this study, we investigated the presence and the transcriptional and metabolic effects of mtDNA variants in EOC PDX cells. Moreover, we unveiled, for the first time, the response of experimental tumors bearing mtDNA variants to bevacizumab, a widely used anti-angiogenic drug in cancer patients.

## Methods

### Ovarian *cancer* patient-derived xenografts

All procedures involving animals and their care adhere to institutional guidelines that comply with national and international laws and policies (EEC Council Directive 2010/63/EU, OJ L 276, 20.10.2010). The animal study was reviewed and approved by the Italian Ministry of Health (Authorization n. 617/2016-PR), in accordance with the Declaration of Helsinki. The xenografts used in this study were established between 2006 and 2017 from ascitic fluids of ovarian cancer patients and have been previously reported [24, 14, 15]. Patient-derived xenografts (PDX), named PDOVCA in this study, were propagated by injecting 1 × 10^6^ tumor cells intra-peritoneally into 8-week-old female NOD/SCID mice (Charles River Laboratories, RRID:IMSR_CRL:394). The animals developed ascitic component at different time points, depending on tumor engraftment and growth. At sacrifice, tumors were harvested by dissection. The ascitic fluid (containing ovarian cancer cells, red blood cells and a small percentage of murine leucocytes) were harvested in phosphate buffered saline (PBS). The biological material derived from the ascitic component (pellets and viable cells) was collected for further assays. Treatment with the anti-human VEGF monoclonal antibody bevacizumab was administered to PDX mice as described in Supplementary Materials and Methods.

### Identification, prioritization and genetic validation of mtDNA variants in PDX cells

Mitochondrial DNA sequencing was performed by Next Generation Sequencing (NGS) methods, as follows. Two long PCR amplicons (9.1 kb and 11.2 kb) were amplified using PrimeSTAR Max DNA Polymerase (Takara Bio, France). The library was constructed by xGen DNA Lib Prep EZ (Integrated DNA Technologies, Inc. Iowa, US) and sequenced paired-end 2 × 150 on MiSeq System (Illumina, San Diego, CA). All the variants called by Mutect2-GATK pipeline are relative to the revised Cambridge Reference Sequence (rCRS, NC_012920). Population frequencies of missense variants and the mtDNA backgrounds on which they were observed were recovered from public database Mitomap (RRID:SCR_002996), checking Genbank, GnomAD and Helix frequency. Haplogroup affiliations of mitogenomes were assigned according to PhyloTree (RRID:SCR_012948). The prioritization of mitochondrial variants was carried out using APOGEE2 prediction tool, available on MitImpact 3D database (mitimpact.css-mendel.it) [13]. Pathogenicity potential was assessed by taking into consideration the frequency in population, the predicted effect on protein/tRNA structure and heteroplasmy. To confirm sequenced mtDNA variants, DNA extracted from both PDX ascites and FFPE patients samples was used to obtain the entire sequence of the mitochondrial genome and analyzed using the rCRS (Ref Seq NC_012920.1). Briefly, 2–5 ng/sample of genomic DNA were amplified with the MitoALL Resequencing kit. PCR products were purified with Multiscreen plates for DNA clean-up (Millipore, #MSNU03050). Direct sequencing of the PCR product was performed with BigDye™ Terminator v1.1 Cycle Sequencing Kit (Thermo Scientific, Waltham, MA, USA). To validate the variants of interest, sequences were run in the ABI 3730 DNA Analyzer for Sanger sequencing. Electropherograms were analyzed with SeqScape® software (Applied Biosystems, Waltham, MA, USA).

### Spectrophotometric determination of respiratory complexes activity

Enzymatic activity of the mitochondrial respiratory chain (mRC) complexes was determined as previously reported [24]. Briefly, crude mitochondria were extracted from 15–50 × 10^6^ PDX cells from ascites. Cell pellet was suspended in ice-cold Sucrose-Mannitol Buffer (200 mM mannitol, 70 mM sucrose, 1 mM EGTA and 10 mM Tris–HCl at pH 7.6) and homogenized using a Potter–Elvehjem homogenizer. Samples were centrifuged at 600 g for 10 min at 4 °C to discard unbroken cells and nuclei. The resulting supernatants were centrifuged at 10,000 g for 20 min at 4 °C to separate crude mitochondria from the remaining sub-cellular fraction. The mitochondria pellet was resuspended in Sucrose-Mannitol Buffer and stored in aliquots at −80 °C. Measurements of respiratory chain complexes activities were assessed at 37 °C in a spectrophotometer (V550 Jasco Europe, Modena, Italy) as previously described [24]. Bradford-based method was used to determine protein concentration. Cells derived from ascitic PDXs either wild type or carrying mtDNA variants with heteroplasmy < 25%, namely PDOVCA 14 (*n* = 1), 15 (*n* = 4), 17 (*n* = 1), 52 (*n* = 2), 145 (*n* = 1), were used as controls. Activity measurements (µM/min) from controls and mutated PDXs with variant load > 50% were normalized to protein concentration (nmol/min x mg) and finally expressed as activity relative to controls (%). At least three independent biological replicates of each PDX were analysed.

### Transmission *electron* microscopy (TEM) and ROS production

Three million cells were harvested from PDX ascites and fixed with 2.5% glutaraldehyde in 0.1 M sodium cacodylate buffer pH 7.4 ON at 4 °C. The samples were postfixed with 1% osmium tetroxide plus potassium ferrocyanide 1% in 0.1 M sodium cacodylate buffer for 1 h at 4 °C. After three water washes, samples were dehydrated in a graded ethanol series and embedded in an epoxy resin (Sigma Aldrich, Missouri, USA). Ultrathin Sects. (60–70 nm) were obtained with a Leica Ultracut EM UC7 ultramicrotome, counterstained with uranyl acetate and lead citrate and viewed with a Tecnai G^2^ (FEI) transmission electron microscope (RRID:SCR_021365) operating at 100 kV. Images were captured with a Veleta (Olympus Soft Imaging System) digital camera. Mitochondrial number and area were measured with ImageJ in 50 images for each PDX and were normalized on the cytosol area of each single image. For ROS measurements, 2 × 10^4^ cells were harvested from PDX ascites and seeded on black 96 well culture plate. The day after, mitochondrial superoxide was measured by using the Fluorometric Mitochondrial Superoxide Detection Kit (Abcam, Cambridge, UK), following manufacturer’s instructions. Control cells were treated with 50 µM Antimycin A (Sigma Aldrich, Missouri, USA) for 1 h. Fluorescence intensity was measured within the VICTOR Nivo Multimode Microplate Reader (PerkinElmer, Massachusetts, USA).

### Histology, immunohistochemistry (IHC) and digital pathology

Cell blocks were obtained by embedding 5 × 10^6^ cells recovered from ascites in agarose (2% w/v). Sequentially, cell blocks were fixed in formalin, embedded in paraffin, and subsequently processed for IHC analysis. Three-micron-thick FFPE tumor samples were stained either with hematoxylin and eosin or processed for IHC. IHC was performed by using anti-MCT4 Rabbit Polyclonal Ab (Santa Cruz Biotechnology, sc-376140, dilution 1:300) and anti-MT-CO1 mouse monoclonal antibody (Abcam Ltd., UK, cat. No. ab14705; dilution 1:5000) through the BOND III and RX automatic stainer (Leica Microsystems, Wetzlar, Germany). The slides were digitally acquired at × 20 magnification by the Aperio CS2 (Leica Biosystems, Wetzlar, Germany; RRID:SCR_025111) and the evaluation of the IHC score was assessed through the Scanscope Image Analysis software (ImageScope v12.4.0.708; RRID:SCR_014311). The signal was analyzed by using the Aperio membrane algorithm v9. For MT-CO1 staining, the slides were digitally acquired at × 20 magnification using the Olympus VS200 Slide Scanner (EVIDENT Life Science). The evaluation of the IHC score was assessed through ImageJ, utilizing the "IHC Profiler" plugin [14].

### RNASEQ pre-processing and analyses

Total RNA was extracted from PDX-derived cells following Trizol® protocol (Life Technologies), according to the manufacturer’s instruction. The sequencing and data analysis methods employed in this study were designed to ensure the accuracy and reliability of the results. All samples underwent 150 paired-end sequencing on Illumina platform. To assess the data quality, FastQC (RRID:SCR_014583) and MultiQC (v.1.14; RRID:SCR_014982) tools were employed. Trimming of the raw reads was carried out using cutadapt (v. 1.18) with specific adapter sequences (AGATCGGAAGAGCACACGTCTGAACTCCAGTCA and AGATCGGAAGAGCGTCGTGTAGGGAAAGAGTGT) and reads below a minimum length of 100 bases were discarded. Subsequently, alignment and quantification of the high-quality human reads against the Gencode GRCh38.p13 reference genome were performed using Salmon (v. 1.4.0; RRID:SCR_017036). The resulting clean and trimmed reads were aligned, and a count matrix was generated in R (v. 4.3.2) with the tximport package. Differential expression analysis was conducted using the DESeq2 package (RRID:SCR_015687) in R, and enrichment analysis was performed using the ClusterProfiler package (RRID:SCR_016884).

### Lactate production and glucose consumption

To characterize the glycolytic metabolism of each PDX, 2 × 10^5^ cells per well in 6-well tissue culture plates were cultured in RPMI 1640 supplemented with 10 mM HEPES (Cambrex Bioscience, East Rutherford, NJ), 1% Sodium Pyruvate (Lonza, Basel, Switzerland), 2 mM L-glutamine and 1% of antibiotic–antimycotic mix (Life Technologies, Paisley, UK), without serum, under normoxic conditions. After 48 h, supernatants were harvested and centrifugated for 10 min at 3000 g and then 1 mL was analyzed considering the basal values present in the medium. Lactate and glucose concentrations in supernatants were determined on an automated analyzer (Cobas 8000, Roche, Basel, Switzerland). Values were normalized to protein content at the end of the incubation period.

### Glucose concentration measurement

Supernatants from ascitic fluids and plasma from blood drawings of PDXs treated or not with bevacizumab were sampled and centrifuged to remove insoluble particles. Glucose concentration was determined using a colorimetric Glucose Assay Kit (Cells Biolabs, Inc., San Diego, CA, US) by following the manufacturer’s instructions. Ascites supernatants were diluted 1:2 for the analysis, whereas plasma samples were diluted 1:100.

### Glucose deprivation assay

For glucose deprivation assay, 5 × 10^4^ cells from selected PDX (PDOVCA 5, PDOVCA 62, PDOVCA 126, PDOVCA 49, PDOVCA 69, PDOVCA 15 and PDOVCA 17) were seeded in 12-well plates both in presence or in absence of glucose. RPMI-1640 Medium with L-Glutamine, without glucose and sodium bicarbonate (Sigma Aldrich, St. Louis, MO, USA) was supplemented with 10% FBS and, when required, glucose was added. The final glucose concentration in glucose-low medium was approximatively 0.036 mg/ml (0.2 mM), whereas in glucose-high medium it was 2 mg/ml (11 mM). After 10 days of culture, cells were detached with trypsin–EDTA and manually analyzed via trypan blue cell viability assay. PDX cells were considered glucose deprivation resistant (GDR) when the ratio: *n*° *of live cells in normal medium* / *n*° *of live cells in low*‑*glucose medium* was higher than the arbitrarily selected threshold of 0.7. Alternatively, if the ratio was below 0.7, PDX cells were considered glucose deprivation sensitive (GDS).

### Statistical analysis

Statistical comparison between two sets of data was performed using the unpaired Student’s *t* test (two-tailed) and ordinary one-way ANOVA with GraphPad Prism (RRID:SCR_002798). Statistical significance is indicated in figure legend as follows: *p < 0.05; **p < 0.01; ***p < 0.001. Data are expressed as mean value ± SD. Survival of mice for in vivo experiments with bevacizumab was evaluated using the Kaplan–Meier method (log–rank test) with SigmaPlot software (RRID:SCR_003210) and survival data were analyzed by the two-tailed Fisher test.

## Results

### Mitochondrial DNA variants are common in EOC PDX cells

**Fig. 1:**
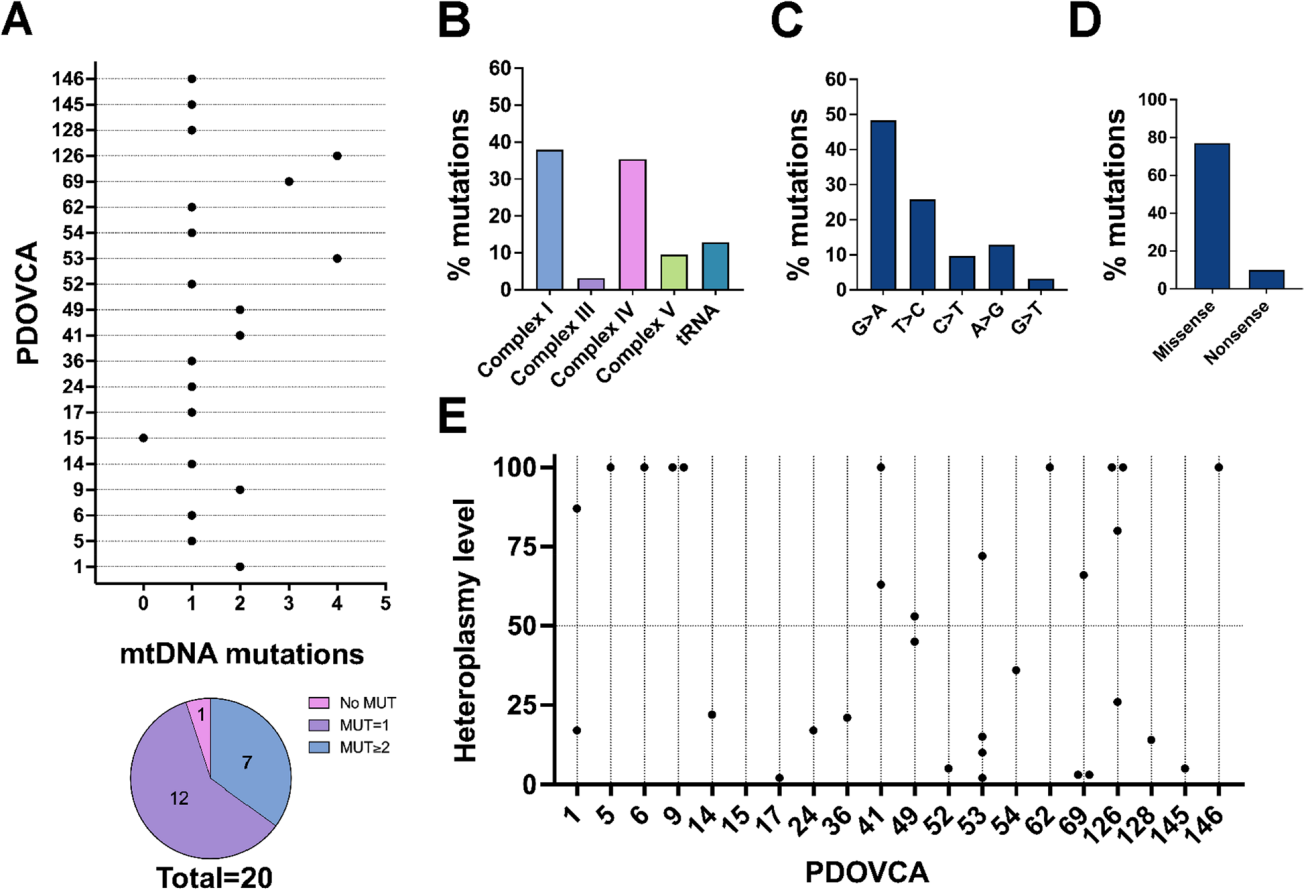
Mitochondrial DNA variants are common in EOC PDX cells.** A** Number of mtDNA variants in EOC PDX (*n* = 20 PDOVCAs). **B** Location of detected mtDNA variants. The percentage of variants occurring in complex I, III, IV and V was calculated on the total number of detected variants. **C **Type of mtDNA point mutation considering base exchange identity. **D** Consequences of mtDNA point mutations on protein translation: proportions of missense and nonsense variants. **E** Heteroplasmy level (%) of each single mtDNA variants in PDOVCAs. Dotted line represents the 50% threshold

To evaluate the mutational status of the mitochondrial genome, we sequenced mtDNA of 20 previously established ovarian cancer PDXs [24, 14, 15]. Altogether, we detected 31 different mtDNA potentially pathogenic variants, with 7 out of 20 PDXs bearing two or more variants, 12 PDXs with one single variant and one PDX without variants (Table 1, Fig. 1A). The highest number of variants (27 out of 31, 87.1%) was found in genes coding for subunits of the OXPHOS complexes, involving Complex I (38%), Complex III (3.2%), Complex IV (35.4%) and ATPase (9.6%), whereas a lower number was observed in tRNAs regions (4 out of 31, 12.9%) (Fig. 1B), reflecting their length within mtDNA molecules (approximately 10%). Variant spectrum analysis showed that G > A (15/31, 48.3%) and T > C (8/31, 25.8%) transitions were the most frequent type of variants (Fig. 1C), suggesting these changes to be likely the result of gamma polymerase errors, rather than subsequent to ROS-induced DNA damage, the former being in fact most common in cancer cells [15]. Among variants that occurred in coding sequences, 77% were missense whereas a small proportion (10%) was nonsense, and no frameshift variants were found (Fig. 1D). Variants were present at various levels of heteroplasmy in PDXs and all but one PDX (PDOVCA 15) carried at least one mtDNA variant (Fig. 1E). To predict a potential damaging effect of these variants on mitochondrial function, we considered as possibly damaging only variants with heteroplasmy above 50% threshold, as proposed by others [13]. Eleven out of 20 PDX (55%) carried at least 1 mtDNA variant at heteroplasmy level > 50% (Fig. 1E and Table 1). Moreover, homoplasmic mtDNA variants were found in 7 out of 20 PDX (35%). When considering the PDXs with more than one variant (*n* = 7), we assumed as principal variant the one at the highest level of heteroplasmy (Fig. 1E, Table 1). To rule out the possibility that the mtDNA mutations found in PDX may have arisen during PDXs propagation, rather than being reminiscent of the original tumor, we exploited all retrievable (*n* = 15) archival (FFPE) specimens of the tumors or ascitic samples collected at diagnosis. In 5 out of 11 original primary solid tumors at least one PDX mutation was detected while, interestingly, nearly all mutations were confirmed in all 4 original ascitic samples analyzed (Suppl. Table S1). This suggested the ascitic cell tumor component to be a better matched control than solid primary for the validation of the maintenance of mtDNA mutations during PDXs propagation, which allowed us to confidently exclude these genetic lesions to be PDX-specific. Additionally, the mean VAF of mtDNA variants confirmed in patients’ samples was 61% ± 34%, whereas the mean VAF of mtDNA variants not confirmed in PDX was 37% ± 38%, indicating that low-heteroplasmy variants are more represented in discordant pairs. The potential co-existence of mitochondrial and nuclear DNA mutations affecting mitochondria components was investigated by whole exome sequencing (WES) in 18 PDOVCAs. By filtering nuclear DNA variants above the 10% VAF threshold and based on the pathogenicity prediction with Varsome classifier, we did not find any pathogenic or likely pathogenic mutations in nuclear genes that encoded for subunits of respiratory complexes (Suppl. Table S2). The only exception was PDOVCA 5, where we discovered the c.364C > T p.(Arg122*) variant (VAF = 32.8%) in *SDHD* which codes for the succinate dehydrogenase complex II subunit D (Suppl. Table S2), along with the homoplasmic stop codon mtDNA variant m.6129G > A/*MT-CO1*.

**Table 1:** List of mitochondrial DNA mutations in PDOVCAs. EnOC: endometrioid ovarian cancer. HGSOC: high-grade serous ovarian cancer. Complex I. subunits ND2, ND3, ND4, ND5, ND6. Complex III: cytochrome b (Cytb). Complex IV: subunits CO1, CO2, CO3, ATPase. MT-TA: mitochondrial transfer alanine. MT-TM: mitochondrial transfer methionine. MT-TV: mitochondrial transfer Valine. NA: not available

Sample ID	OC Histotype	Aplo mtDNA	mtDNA mutations	aa change	Mutation prediction	Heteroplasmy VAF
**PDOVCA 1**	HGSOC	H58	m.7763G > A/*MT-CO2*	p.E60K	Pathogenic	17%
			m.9412G > A/*MT-CO3*	p.G69D	Pathogenic	87%
**PDOVCA 5**	HGSOC	U5a2b	m.6129G > A/*MT-CO1*	Stop	Stop	100%
**PDOVCA 6**	HGSOC	K1a4d	m.6691G > A/*MT-CO1*	p.G263E	Pathogenic	100%
**PDOVCA 9**	HGSOC	H3	m.4686G > A/*MT-ND2*	p.A73T	Pathogenic	100%
			m.5591G > A/*MT-TA*		Pathogenic	100%
**PDOVCA 14**	EnOC	X4	m. 13180G > A/*MT-ND5*	p.A282T	Pathogenic	22%
**PDOVCA 15**	HGSOC	V18	Wild type	wt	wt	-
**PDOVCA 17**	HGSOC	U5a1g1	m.11841 T > C/*MT-ND4*	p.L361P	Pathogenic	2%
**PDOVCA 24**	HGSOC	H41a	m.8759 T > C/*MT-ATP6*	p.F78S	Pathogenic	17%
**PDOVCA 36**	HGSOC	H59	m.10197G > A/*MT-ND3*	p.A47T	Pathogenic	21%
**PDOVCA 41**	EnOC	H6a1a9	m.6589 T > C/*MT-CO1*	p.I229T	Pathogenic	63%
			m.8538 T > C/*MT-ATP8*	p.I58T	Pathogenic	100%
**PDOVCA 49**	HGSOC	U3b3	m.1666 T > C/*MT-TV*	-	Likely polymorphic	45%
			m.6745G > A/*MT-CO1*	p.G281D	High pathogenicity	53%
**PDOVCA 52**	HGSOC	U5b1	m.11868C > T/*MT-ND4*	p. P370L	High pathogenicity	5%
**PDOVCA 53**	EnOC	H1e1	m.4558G > A/*MT-ND2*	Stop	Stop	2%
			m.6126A > G/*MT-CO1*	p. I75V	Pathogenic	72%
			m.7030A > G/*MT-CO1*	p. H376R	Pathogenic	10%
			m.8529G > T/*MT-ATP8,ATP6*	p. W55L, p. M1I	Pathogenic	15%
**PDOVCA 54**	HGSOC	R0a2f	m.4142G > A/*MT-ND1*	p.R279Q	Pathogenic	36%
**PDOVCA 62**	HGSOC	H56a	m.14958A > G/*MT-CYB*	p.R71Q	Pathogenic	100%
**PDOVCA 69**	HGSOC	J1d3a2	m.3481G > A/*MT-ND1*	p.E59K	Pathogenic	3%
			m.3811C > T/*MT-ND1*	Stop	Stop	3%
			m.6870T > C/*MT-CO1*	p.W323R	Pathogenic	66%
**PDOVCA 126**	HGSOC	H1cg	m.6340C > T/*MT-CO1*	p.T146I	Pathogenic	100%
			m.13759G > A/*MT-ND5*	p.A475T	Pathogenic	80%
			m.14693A > G/*MT-TE*	-	Not Known	100%
			m.13048G > A/*MT-ND5*	p.E238K	Pathogenic	26%
**PDOVCA 128**	HGSOC	X2l	m.4449G > A/*MT-TM*	-	Likely polymorphic	14%
**PDOVCA 145**	HGSOC	H6a1b4	m.13946T > C/*MT-ND5*	p.I537T	Pathogenic	5%
**PDOVCA 146**	HGSOC	K1a19	m.614180T > C/*MT-ND6*	p.Y165C	NA	100%

### mtDNA variants affect mitochondrial ultrastructure and OXPHOS complexes activity and abundance

**Fig. 2:**
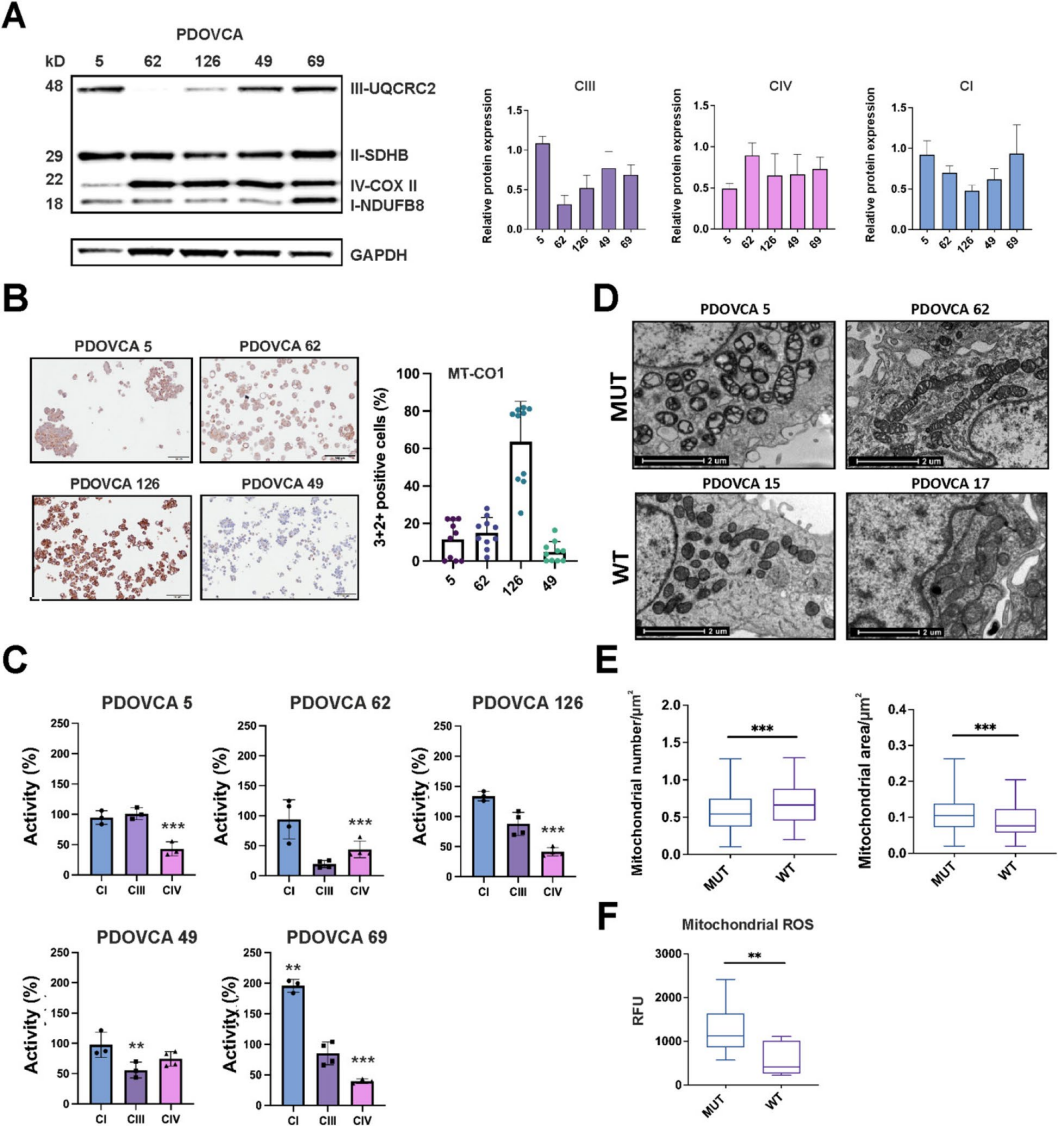
mtDNA variants affect mitochondrial ultrastructure and OXPHOS complexes activity and abundance. **A** Representative western blot analysis and relative protein expression of mitochondrial complexes subunits (UQCRC2, SDHB, COXII, NDUFB8) in PDOVCAs. GADPH was used as loading control and to perform densitometric normalization. **B** Representative images and relative quantification of MT-CO1 protein expression were obtained by IHC in PDX cells. The percentage of 3 + 2 + positive cells was measured through ImageJ. Scale bar 100 µM. **C** Activity of mitochondrial respiratory complexes determined by spectrophotometric kinetic measurements and normalized to protein concentration in homoplasmic (VAF = 100%, PDOVCA 5, 62, 126) and heteroplasmic (VAF < 100%, PDOVCA 69 and 49) PDOVCAs. Data (n ≥ 3) are mean ± SD and expressed as percentage relative to controls (wild type and mutated PDOVCA with heteroplasmy < 25%). *p < 0.05, **p < 0.01, ***p < 0.001, according to ordinary one-way ANOVA. **D** Representative TEM images of mutated (MUT: PDOVCA 5, 62) and wild-type (WT: PDOVCA 15, 17) PDOVCA-derived cells. Cells were collected, fixed, embedded, sectioned and viewed using TEM. Mutated mitochondria were visualized and appeared morphologically altered respect to wild-type ones. Scale bar 2 µM. **E** Mitochondrial number and area were calculated from TEM images (*n* = 50) in MUT and WT PDOVCAs. Both parameters were obtained normalizing the number of mitochondria and the occupied area on cytosol area in each image. **F** Mitochondrial ROS in MUT and WT PDOVCAs. Data (n ≥ 3) are mean ± SD (*n* = 3 biological replicates for each PDOVCA). **p < 0.01, ***p < 0.001, according to unpaired Student’s *t* test (two-tailed)

To evaluate the functional impact of mtDNA variants predicted as pathogenic we investigated a subset (*n* = 10) of PDXs with different mtDNA genotype status: 3 homoplasmic mutated PDXs (PDOVCAs 5, 62, 126), 2 heteroplasmic mutated PDXs (heteroplasmy > 50%: PDOVCA 49, 69) and 5 control PDXs (PDOVCAs 14, 15, 17, 52, 145) (Table 1). Functional investigations included assessment of mRC complexes abundance through the evaluation of essential subunits and enzyme activity. PDOVCA 5 cells bore the homoplasmic stop codon variant m.6129G > A/*MT-CO1*, which is predicted to generate a truncated protein of 76 amino acids, thus affecting the stability and abundance of MT-CO1. Accordingly, we observed a reduction of the steady state levels of two core subunits of CIV, namely MT-CO1 and MT-CO2 by western blot analyses of total lysates and isolated mitochondria (Fig. 2A and Suppl. Fig. S1). Moreover, IHC analyses showed MT-CO1 positive score mean of 11.5± 3.19% (Fig. 2B). Lastly, a decrease of about 50% of CIV activity was found in comparison to controls (Fig. 2C). PDOVCA 62 carried the homoplasmic missense variant m.14958G > A/*MT-CYB* which induced the amino acid substitution p.Arg71Gln in a conserved region of the only subunit of CIII encoded by mtDNA (cyt *b*), which has never been described before in cancer. This variant induced a dramatic decrease of CIII activity (Fig. 2C) and PDOVCA 62 cells showed also a reduction in UQCRC2 levels (Fig. 2A), which may derive from an alteration of CIII stability or assembly. Intriguingly, PDOVCA 62 showed also a mild reduction of CIV activity (Fig. 2C) and a reduced positivity for MT-CO1 in IHC (MT-CO1 positive score mean of 15.1± 2.58%) (Fig. 2B), possibly indicating a secondary CIV defect and highlighting an interdependence of CIII and CIV [24, 14, 15]. PDOVCA 126 harbored four mtDNA variants, namely homoplasmic m.6340C > T/*MT-CO1* and m.14693A > G/*MT-TE*, heteroplasmic m.13759G > A/*MT-ND5* (variant load 80%) and low-heteroplasmic m.13048G > A/*MT-ND5* (variant load 26%) (Table 1). Considering the high variant load, the first three variants were predicted as possibly pathogenic. However, the determination of respiratory complexes activities showed an isolated CIV impairment (Fig. 2B) indicating the m.6340C > T/*MT-CO1* missense variant as the most detrimental. Both MT-CO1 and MT-CO2 were expressed (MT-CO1 positive score mean of 63.65± 6.79%) (Fig. 2A,B), indicating that CIV stability was not affected. In particular, IHC showed that MT-CO1 reached the highest levels in PDOVCA 126 compared to PDOVCAs 5 and 62 (Fig. 2B). Since *MT-CO1* and *MT-CO2* are mtDNA encoded, their high abundance suggests that the m.14693A > G/*MT-TE* does not exert a functional effect on mitochondrial protein synthesis. PDOVCA 49 presented with the co-occurrence of two heteroplasmic mtDNA variants (variant load range 45–53%), namely m.1666 T > C/*MT-TV* and the missense m.6745G > A/*MT-CO1*. This sample displayed negative MT-CO1 staining in IHC (MT-CO1 positive score mean of 4.94± 1.70%) (Fig. 2B). Furthermore, assessment of mRC complexes activities revealed a reduction of CIII and CIV function (Fig. 2C) that may derive from the combination of the two heteroplasmic variants and/or due to the previously mentioned interdependence of these two enzymes, with a secondary CIII defect caused by a variant in *MT-CO1*. Lastly, PDOVCA 69 harbored only one potentially pathogenic mtDNA variant, i.e. the heteroplasmic m.6870 T > C/*MT-CO1* (variant load 66%). This variant induced a significant decrease in CIV activity (Fig. 2C). It is important to note that PDOVCA 69 showed an increased CI activity that may be suggestive of an attempt of these cells to compensate the downstream OXPHOS dysfunction induced by the m.6870 T > C/*MT-CO1* variant. These results indicate that mtDNA variants, both homoplasmic and heteroplasmic with a variant load > 50%, affect the mRC function, allowing us to cluster them together despite the individual differences. The presence of mtDNA variants in cancer cells has been associated with aberrant mitochondria morphological properties and increased ROS production [13, 24, 14, 15]. Determination of mitochondrial ultrastructure by TEM revealed that mitochondria of mutated PDOVCAs, except for PDOVCA 49 which had morphologically diverse mitochondria, were irregularly shaped, large, swollen and endowed with enlarged electron-dense *cristae* when compared to WT ones (PDOVCA 15, 17), corroborating the underlying mitochondrial dysfunction (Fig. 2D)*.* Moreover, mutated PDOVCAs had a lower number of larger mitochondria than WT PDOVCA which oppositely presented a higher number of smaller mitochondria (Fig. 2E). To further strengthen our results, mutated PDXs produced a higher amount of mitochondrial ROS compared with WT ones (Fig. 2F), indicating that these variants impact on respiratory complexes function and induce a chronic oxidative stress in PDOVCA cells. All these results demonstrated that mtDNA variants were able to trigger metabolic stress in EOC cancer cells and to affect mitochondria ultrastructure.

### mtDNA variants induced transcriptional deregulation of metabolic-related processes in PDOVCAs

**Fig. 3:**
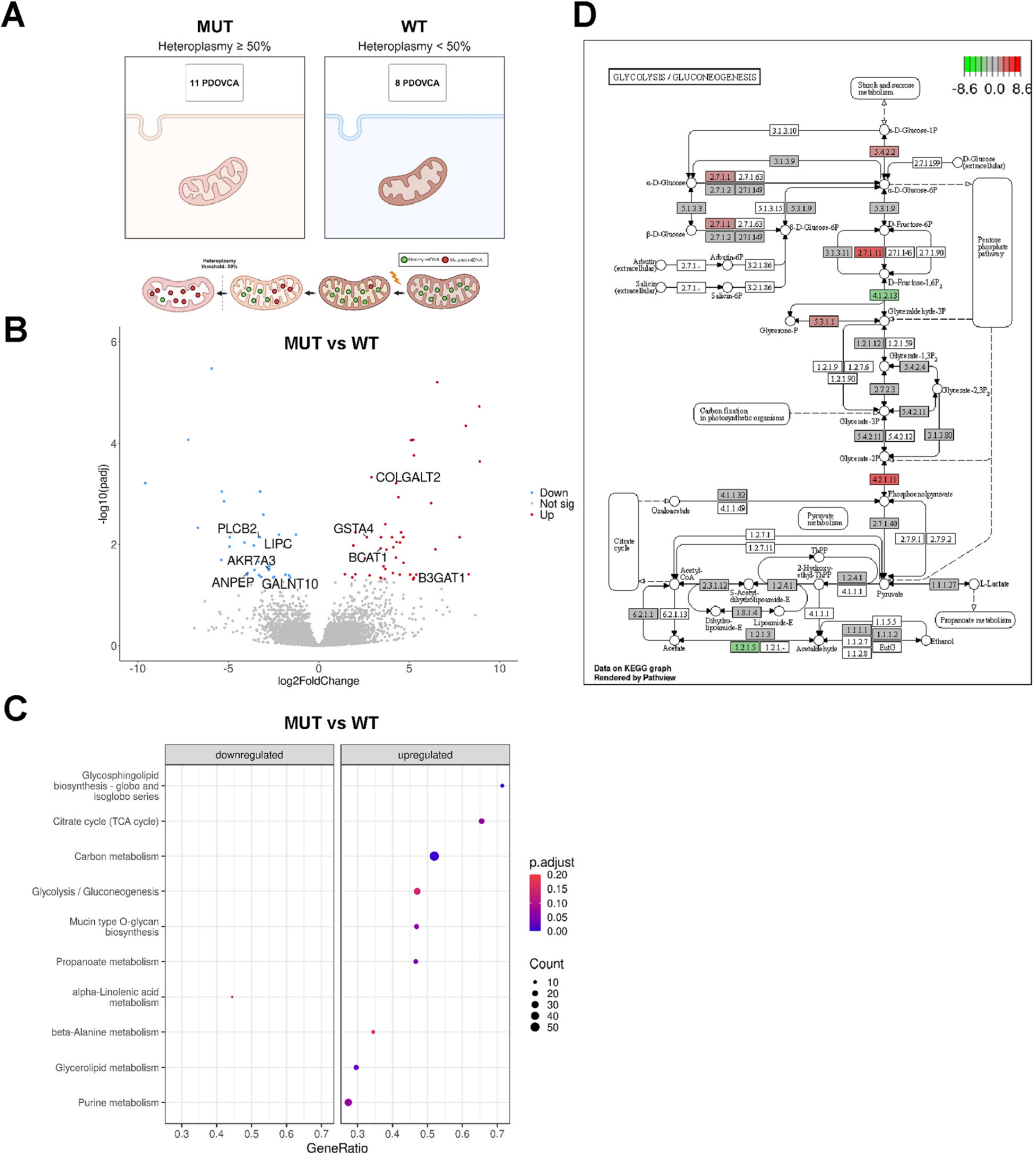
mtDNA variants induced the transcriptional deregulation of metabolic-related processes in PDXs. **A** For differential analysis, PDOVCAs (*n* = 19) were classified as either mutated (MUT = heteroplasmy > 50%, *n* = 11) or wild type (WT = heteroplasmy < 50%, *n* = 8). **B **Volcano plot, the Log2FoldChange indicates the fold change in gene expression between the two conditions (MUT vs. WT). Each dot represents one gene: grey genes represent non-significant DEGs between mutated (MUT) vs wild type (WT), the red dots represent up-regulated genes and the blue ones represent down-regulated genes. **C** GSEA enrichment plots showing significantly upregulated (*n* = 9) and downregulated (*n* = 1) KEGG pathways in MUT vs. WT PDOVCAs. Bubble colours represent the p.adj (blue = most significant), bubble size represents the number of genes enriched, gene-ratio indicates the degree of enrichment for the corresponding gene set. **D **KEGG pathway graph rendered by Pathview. KEGG Pathview showing Glycolysis/Gluconeogenesis pathway genes up (red) or down (green)-regulated in MUT versus WT PDOVCAs

To investigate whether mtDNA variants were associated with peculiar signatures at the transcriptional level, we arbitrarily classified PDOVCAs (*n* = 19) as either mutated (MUT, *n* = 11) or non-mutated (WT, *n* = 8) according to the mutation load of pathogenic mtDNA variants by using heteroplasmy = 50% as cutoff value (Fig. 3A). RNA-seq analysis was conducted to unveil differences in transcriptional modulations between the two groups that could potentially be associated with mtDNA variants. Gene expression analysis disclosed 84 differentially expressed genes (DEGs), of which 36 and 48 were significantly down- and up-regulated, respectively (Fig. 3B, Suppl. File 1). We observed that among DEGs, 9 belonged to metabolic pathways, such as *ANPEP, GALNT10, AKR7A3, LIPC, PLCB2, B3GAT1, BCAT1, COLGALT2* and *GSTA4* (Fig. 3B). To explore the functional implications of mtDNA variants, we conducted gene set enrichment analysis on KEGG pathways. Several pathways were deregulated in mutated PDXs compared with wild type ones, including, among others, PIK3CA-Akt signaling, MAPK signaling, AMPK and HIF1-α pathways, which have been implicated in mitochondrial dysfunction [13, 24] (Suppl. File 1). With regard to metabolic pathways, among the 189 KEGG metabolism-related processes analyzed, we found 10 metabolic pathways that were significantly deregulated. Upregulated pathways in mtDNA mutated PDX included glycosphingolipid biosynthesis-globo and isoglobo series, citrate cycle (TCA cycle), carbon metabolism, glycolysis/gluconeogenesis, mucin type O-glycan biosynthesis, propanoate metabolism, beta-Alanine metabolism, glycerolipid metabolism and purine metabolism. In contrast, alpha-linolenic acid metabolism was downregulated (Fig. 3C). Although all the upregulated metabolic pathways are potentially relevant for tumor biology [14, 44, 15, 48, 13, 24, 14, 44], given the established role of the Warburg effect in cancer, we focused on the genes involved in deregulation of glycolysis/gluconeogenesis (hsa00010) (Fig. 3D). Five genes (*PGM1, HK1, PFKL, TPI1, ENO1*) were activated, whereas two were suppressed (*ALDOA* and *ALDH3A1*) indicating broad multi-level modulation of this pathway (Fig. 3D). We delved also into the principal common deregulated genes between the strictly glycolysis-related TCA cycle and glycolysis pathways in mutated PDOVCAs, by finding *PCK1, PCK2*, *DLD* and *ACO1* as the interconnected genes between the two pathways (Suppl. Figure S2, S3). All these results suggest that certain mtDNA variants may underlie a potential induction of a metabolic rewiring in tumor cells, ultimately enhancing glycolysis to favor the Warburg effect.

### mtDNA variants triggered a phenotypic metabolic rewiring toward glycolysis in PDOVCAs

**Fig. 4:**
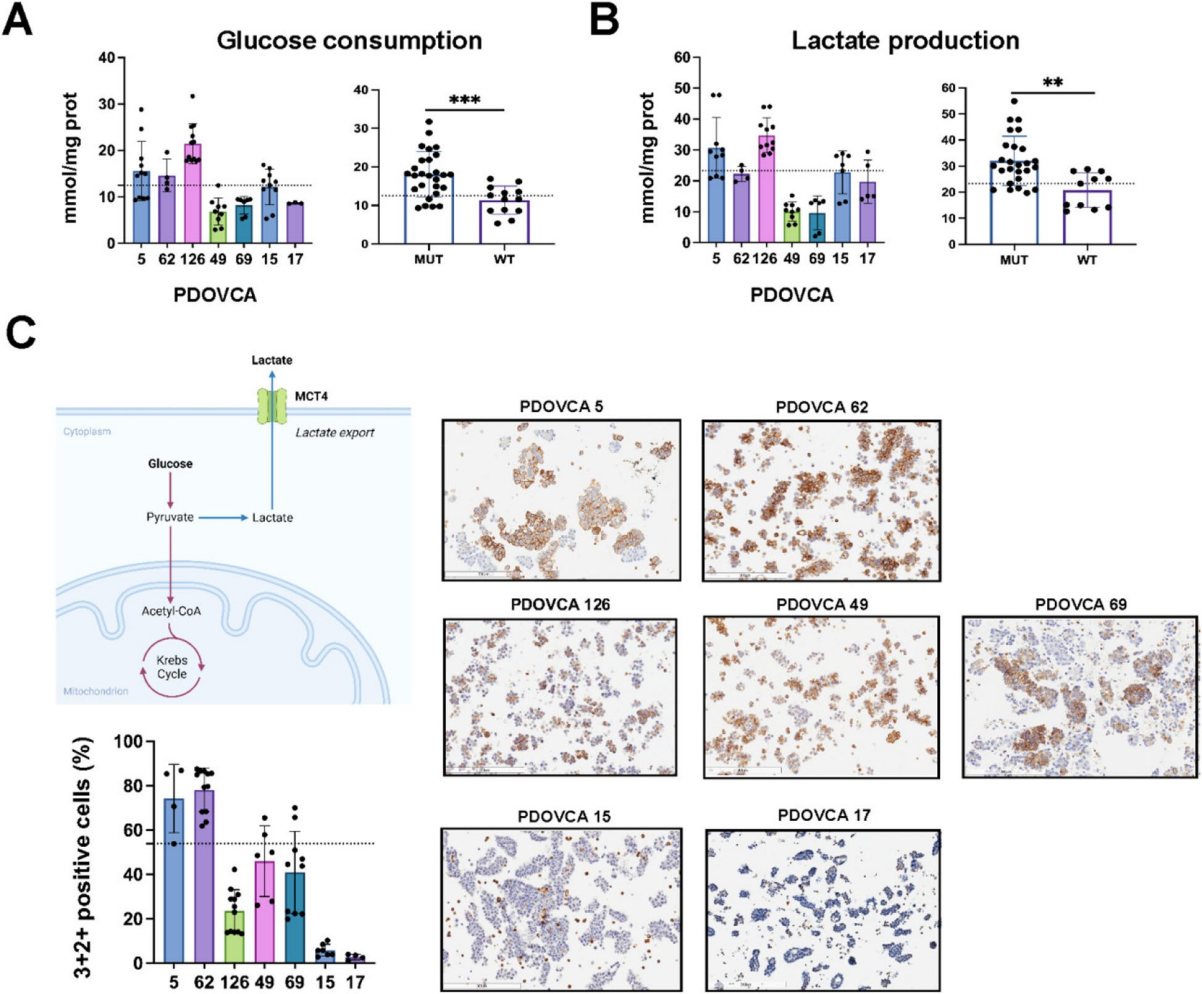
mtDNA variants triggered a phenotypic metabolic rewiring towards glycolysis in PDXs.** A** Glucose consumption and **(B)** lactate production rate in PDOVCAs after 48 h cultured in vitro. Right panel: PDOVCAs were grouped in MUT (heteroplasmy ≥ 50%) and WT (heteroplasmy < 50%). Dotted lines represent the threshold based on the median of measurements for each PDOVCA. **C** Monocarboxylate transporter 4 (MCT4) in cancer cells: lactate is excessively produced in cancer cells within glycolysis and, to overcome excessive acidification, is extruded by MCT4 lactate transporter (produced by BioRender). Right panel: representative images and relative quantification of MCT4 protein expression were obtained by IHC in PDOVCAs. The percentage of 3 + 2 + positive cells was measured by digital pathology analysis and PDOVCAs were classified MCT4^high^ or MCT4.^low^ based on the median threshold (53.90%). Scale bar 300 µM. **p < 0.01, ***p < 0.001, according to unpaired Student’s *t* test (two-tailed)

Our findings indicated that several mtDNA variants were associated with a glycolysis-related transcriptional signature, reduction of expression of OXPHOS complexes at the protein level as well as reduced enzymatic activity. To further characterize the metabolic phenotype of PDX cells and correlate it with mtDNA variants, we measured glucose consumption and lactate production in vitro. By establishing the median glucose concentration value (M = 12.48 mmol/mg protein) as a threshold, PDOVCAs were classified either as highly (mmol/mg protein > M), or poorly glucose consuming (mmol/mg protein < M). Likewise, the median lactate concentration value (M = 23.29 mmol/mg protein) was used to classify PDOVCAs as highly or poorly lactate producing PDXs (Fig. 4A). Based on this cut-off, PDOVCA 5, PDOVCA 62 and PDOVCA 126 were classified as highly glucose consuming and lactate producing PDXs (Fig. 4B). By observing that PDX cells produced different amounts of lactate in vitro, we hypothesized that its membrane transporter [14] could be diversely expressed. To tackle this, protein expression of the glycolysis-associated monocarboxylate transporter 4 (MCT4) (Fig. 4C) was evaluated by IHC and the percentage of 3 + 2 + cells derived from ascites was used for classification into MCT4^high^ and MCT4^low^ expressing PDOVCAs, by using median value (53.90%) as threshold (Suppl. Figure S4 A,B). Among the seven PDOVCAs that we characterized, we found that the highest MCT4 protein expression level was present in PDOVCA 5 and PDOVCA 62 cells, whereas PDOVCA 15 and PDOVCA 17 cells exhibited the lowest MCT4 levels, and PDOVCA 49, 126 and 69 had intermediate MCT4 expression levels (Fig. 4C). At the transcript level, MCT4 mRNA level was higher in PDOVCA 5, 62 and 126 compared to PDOVCA 49, 69, 15 and 17 (Suppl. Figure S4 C). Altogether, these results indicate that homoplasmic mutated PDOVCAs have a metabolic preference for glycolysis suggesting that mtDNA variants were able to directly impact on metabolic rewiring of PDX cells.

### mtDNA variants conferred a survival advantage to anti-VEGF therapy treated mice

**Fig. 5:**
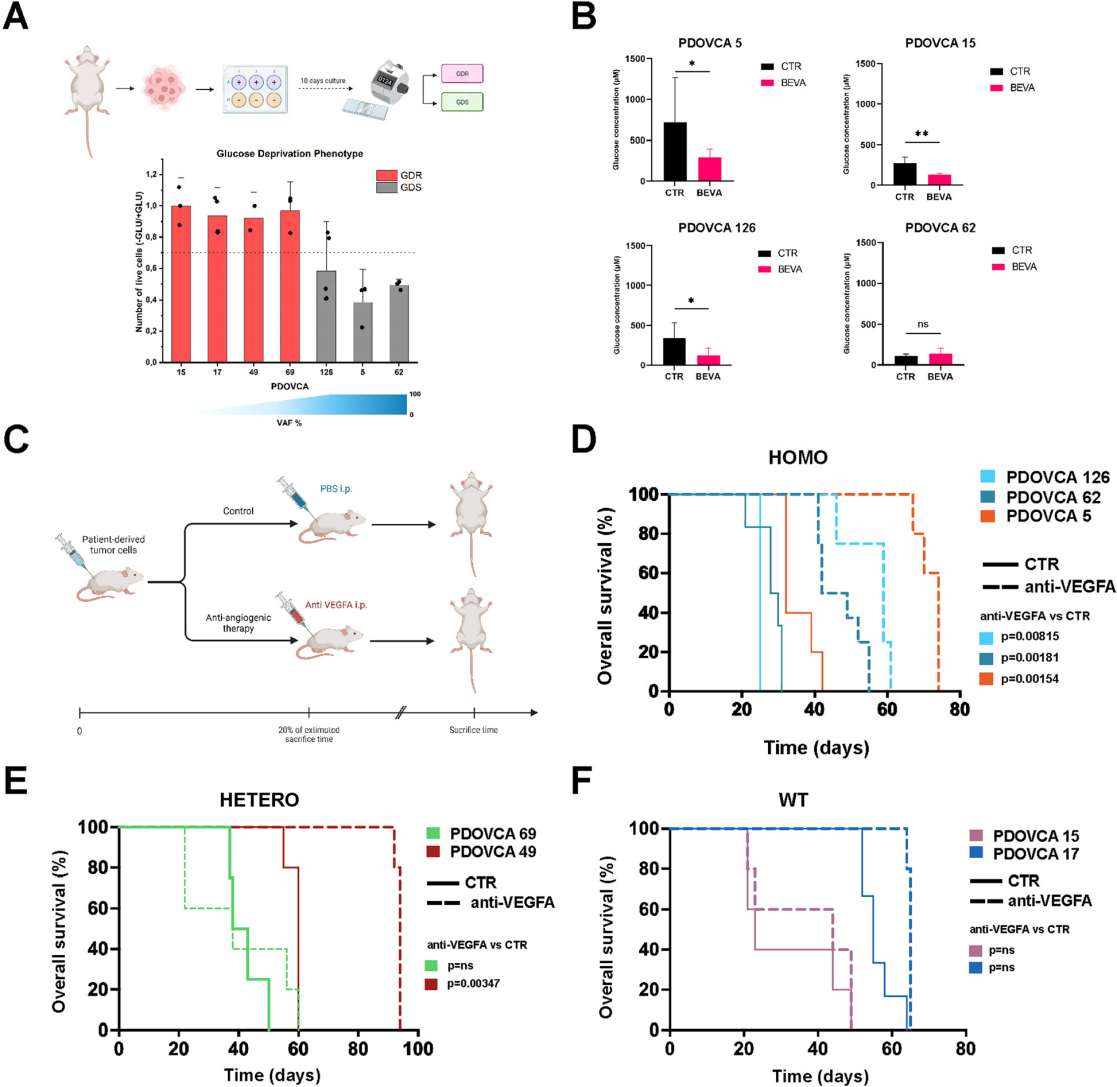
Homoplasmic mtDNA mutations associate with survival advantage when bevacizumab was administered to PDOVCA.** A** Glucose deprivation assay in vitro: PDOVCA-derived cells were maintained in culture upon glucose deprivation for 10 days and then vitality was measured with trypan blu exclusion method. GDS and GDR phenotype was assigned based on the number of (–) glu cells on ( +) glu cells, with GDS < 0.7 and GDR > 0.7. **B** Glucose concentration measurement in CTR and short-term anti-VEGF (BEVA) treated PDOVCA-derived ascites of PDOVCA 5 (controls *n* = 3, treated *n* = 8), PDOVCA 15 (controls *n* = 4, treated *n* = 5), PDOVCA 126 (controls *n* = 4, treated *n* = 7) and PDOVCA 62 (controls *n* = 3, treated *n* = 7)**. C** For long term treatment experiments, 1 × 10^6^ cells of PDOVCA 5, 62, 126, 49, 69, 15 and 17 (*n* = 5 mice for the control group and *n* = 5 as treated group) were intra-peritoneally injected in NOD/SCID mice and, depending on the growth time of the PDX models, we started the intraperitoneal treatment with anti-VEGF at 20% of the estimated time to sacrifice until ascites formation was observed. Control mice received intraperitoneal injections of PBS. At sacrifice, ascitic fluid containing cancer cells and blood samples were collected to perfom further assays. **D-F** Kaplan–Meier curves of PDOVCAs treated with long-term anti-VEGF drug bevacizumab in vivo. Homoplasmic mutated PDOVCAs (5, 62, 126), heteroplasmic mutated PDOVCAs (49, 69) and wild-type PDOVCAs (15, 17) were depicted. Log-rank statistical analysis was performed

As a further potential indicator of the metabolic preference for glycolysis in some PDOVCAs [56], we sought to measure the level of glucose addiction in PDX cells in vitro. The effect of glucose deprivation was evaluated by maintaining PDX cells under low-glucose culture conditions in vitro. According to cell viability assay at the endpoint, as described in the Materials and Methods section, PDOVCAs were classified either as Glucose Deprivation Resistant (GDR), or Glucose Deprivation Sensitive (GDS). PDOVCA 5, PDOVCA 62 and PDOVCA 126 were characterized by a GDS phenotype because they did not survive under low glucose conditions. Conversely, PDOVCA 49, 69, 15 and 17 were defined as GDR due to their relatively better survival under glucose starvation (Fig. 5A). Notably, glucose deprivation assays were previously performed with tumor cells deriving from the ascitic fluid of 4 EOC patients who were also donors of the tumor cells used to establish PDX. Based on results of the assay, patients’ tumor cells were classified as GDS or GDR [15]. Importantly, a substantial match in terms of GDS/GDR phenotype was observed between patients’ tumor cells and the matched PDX cells (Suppl. Table S3), indicating that some metabolic features are conserved between patient-derived and PDX tumor cells.

Starting from previously published data indicating that anti-VEGF therapy decreased glucose levels in subcutaneous xenografts of ovarian cancer cell lines [12], we subsequently investigated whether short-term treatment with the antiangiogenic drug bevacizumab altered glucose concentration in the PDOVCA’s ascites in vivo. To evaluate the effect of bevacizumab on glucose concentration in ascitic fluids, we performed an in vivo experiment with four different PDOVCA models: PDOVCA 5, 15, 126 and 62. For each model, ascites and plasma samples glucose concentration were determined in the control group compared to bevacizumab treated group. The mean glucose concentration measured in murine plasma samples was 5282 μM, comparable to the values previously reported in the literature [57]. Mean glucose concentration in control mice ascitic fluid was substantially lower than in plasma and rather heterogeneous, ranging from 111.2 to 715.5 μM. Interestingly, glucose concentration in the ascitic fluid of treated mice of PDOVCA 5, PDOVCA 15 and PDOVCA 126 mice was significantly lower compared with the control group. In one model, PDOVCA 62, no changes were observed in glucose concentration between the two groups, although this PDX model was characterized by markedly low glucose levels at baseline, compared with other PDX models (Fig. 5B). Finally, since bevacizumab was able to induce glucose deprivation in ascites of PDOVCAs, and some PDX cells exhibited marked vulnerability when treated under glucose starvation in vitro, we sought to investigate the potential impact of mtDNA variants and the correlated metabolic alterations on response to anti-angiogenic treatment (Fig. 5C). Initially, we analyzed the impact of mtDNA variants on tumor growth when tumors were grown i.p. as PDXs without any treatment and we did not observe significant differences in survival between mtDNA mutated and not mutated PDXs, with similar growth rates between the two groups (MUT = 36.2 ± 13.9 days; WT = 38 ± 14.4 days) (Suppl. Figures S5). However, in several PDX models we observed that homoplasmic mtDNA variants correlated with a survival advantage following bevacizumab treatment (PDOVCA 5: CTR 35.4 ± 4.7 days vs anti-VEGFA 71.8 ± 3.1 days; PDOVCA 62: CTR 28.7 ± 3.7 days vs anti-VEGFA 47.1 ± 6.3 days, PDOVCA 126: CTR 25 days vs anti-VEGFA 53 ± 8.1 days; PDOVCA 6: CTR 17.7 ± 3.4 days vs anti-VEGFA 28 ± 1.4 days; PDOVCA 41: CTR 50.8 ± 4 days vs anti-VEGFA 76.4 ± 9.5 days) (Fig. 5D and Suppl. Figure S6). On the other hand, PDX bearing heteroplasmic mtDNA variants disclosed heterogeneous response to anti-VEGF treatment, with PDOVCA 49 responding to treatment and PDOVCA 69 lacking any response to treatment (Fig. 5E). Finally, wild-type PDXs did not benefit from anti-VEGF treatment (Fig. 5F). These results indicate that homoplasmic mtDNA variants are associated with a significant survival advantage upon anti-angiogenic treatment in PDX models (two-tailed Fisher test, *p* = 0.0476).

## Discussion

Mitochondrial DNA variants have been reported in many tumor types, occasionally at high heteroplasmy level or homoplasmy [15], but their possible impact on anti-cancer therapy is still largely uncharted. Recently, it has been reported that pathogenic mtDNA mutations inactivating complex I correlate with response to immune checkpoint blockade in mouse models of melanoma and in a cohort of melanoma patients treated with the anti-PD1 monoclonal antibody nivolumab [14]. However, to the best of our knowledge, there was no information about the possible impact of mtDNA mutations on the effects of anti-angiogenic therapy. We considered this an interesting area of investigation because on one hand the potential of mtDNA mutations to alter metabolic pathways in cancer cells is well established and on the other hand we and others previously showed that anti-VEGF therapy causes severe perturbations in oxygen levels and nutrients in the tumor microenvironment [15]. To tackle this in pre-clinical models, we characterized mtDNA mutations in EOC PDX, a type of cancer which is currently treated with anti-VEGF drugs, in combination with chemotherapy and other drugs such as PARP inhibitors [3]. In ovarian cancer patients, mtDNA variants have been described in a substantial fraction (36%) of tumor samples [15, 13, 48] and have been associated with resistance to standard chemotherapy and disease recurrence [57, 57]. Here, we found that the large majority of EOC PDX tested (19 out of 20, 95%) bear mtDNA variants at different levels of heteroplasmy in the coding sequences of respiratory complexes. This is true also if we consider only mtDNA variants at > 50% VAF, which were found in 55% of the PDX samples analyzed. Notably, as the PDX samples do not contain human stromal cells, these mtDNA variants should be considered tumor-specific, although it is known from the literature that normal cells may also accumulate mtDNA mutations, especially during aging [13]. The numbers of mtDNA mutations described here in PDX samples are higher than those previously reported in EOC patients [15, 13]. A potential explanation for this may be hinted by our findings that the liquid phase of the tumor may better mirror the mitochondrial genotype than the solid primary tumor. Indeed, although mtDNA mutations are detected also in nearly half original tumor specimens, the inherent heterogeneity of sampling indicates that the ascitic component maintains more often such genetic lesions. It is tempting to speculate that mtDNA-mutated subpopulations of cancer cells, underrepresented in the solid tumor sampling, may detach and be fit in the liquid phase, where they may thrive and expand, whereby PDXs represent the ideal propagating environment. Additional research is warranted on larger cohorts of samples to validate this hypothesis and delve into the causes for such selection in ascites. Most mtDNA variants found were transitions (Fig. 1C), suggesting that they could be due to POLG errors rather than being ROS-induced, which are mainly associated with G > T transversions [57]. However, we detected increased mitochondrial ROS levels in selected PDX bearing homoplasmic mtDNA mutations (Fig. 2F), suggesting that increased ROS could be the consequence rather than the cause of these mutations. These results are also in line with the conclusions of previous studies which characterized ROS-generating mtDNA mutations and described their role in the promotion of tumorigenesis and metastasis [13, 24].

Based on our previous findings in tumor xenografts [12], showing that glycolytic tumors could better respond to antiangiogenic therapy, our working hypothesis was that certain variants could interfere with the physiological activity of mitochondrial complexes thus producing a distinct glycolytic metabolic signature, and tested whether these metabolic traits might render cancer cells more sensitive to anti-VEGF therapy in experimental tumor models. Homoplasmic mtDNA variants, predicted to be pathogenic, significantly impacted mitochondrial respiration in mutated PDXs, leading to a marked upregulation of glycolysis, in line with observations by other groups [14]. This shift resulted in a Warburg-like phenotype, making these tumor cells 'glucose addicted'. This was confirmed by in vitro glucose starvation assays, which highlighted a metabolic vulnerability under metabolic stress of PDX cells bearing pathogenic homoplasmic mtDNA variants. This 'glucose addicted' phenotype may result from the reduced metabolic flexibility of tumor cells imposed by homoplasmic mtDNA variants. Could this metabolic phenotype also promote tumorigenesis? While we have not investigated this further, we speculate that tumor cells with pathogenic mtDNA mutations, which detach from the primary tumor and disseminate into the ascitic fluid, may undergo positive selection in this liquid tumor environment, where upregulated glycolysis may represent an advantage given the distance of tumor cells from the blood vessels [14].

The key finding of our study was the correlation between homoplasmic pathogenic mtDNA mutations and survival of the mice following treatment with bevacizumab. Importantly, this correlation was found also in PDX models bearing mtDNA mutations confirmed also in the matched patient’s DNA. Which is the hypothetical mechanistic explanation of this effect? Anti-VEGF therapy using a monoclonal antibody neutralizing VEGF-A was known to cause severe glucose deprivation in subcutaneous xenografts of ovarian cancer, as shown by induced metabolic bioluminescence imaging [12]. Although the TME of PDX models is substantially different compared with that of subcutaneous tumors, we confirmed these effects of antiangiogenic therapy in 3 PDX models. We thus speculate that anti-VEGF therapy could lead to improved tumor control when administered to “glucose-addicted” PDX bearing pathogenic and homoplasmic DNA variants due to its effects on nutrients availability, and especially glucose, in the tumor microenvironment. In this regard, it is interesting to remark that patients’ tumor cells in the ascitic fluid and matched PDX cells disclosed similar GDR/GDS phenotypes under glucose starvation (Suppl. Table S3).

Anti-angiogenic treatment with bevacizumab has demonstrated some therapeutic benefits in combination with the standard chemotherapy for advanced EOC [44] but in the absence of predictive biomarkers of response clinical results, especially in terms of overall survival, have been modest [7]. It is striking to note that colorectal cancer, one of the few tumors where bevacizumab had clear-cut positive therapeutic effects in terms of survival [56], is the tumor type with the highest frequency of mtDNA variants [15, 15].

These provoking preclinical findings have potential translational implications, but we acknowledge that PDX models have some intrinsic limitations and many open questions will require substantial work to be addressed in clinical studies. First, it will be fundamental to assess the percentage of EOC patients bearing homoplasmic mtDNA mutations, which cannot be accurately obtained from public datasets. Related to this, is there accumulation of mtDNA variants also in stromal cells of human tumors and do they also contribute to the modulation of nutrients availability in the tumor microenvironment? Furthermore, is there any effect of heteroplasmic (> 50%) mutations on bevacizumab response in patients and which is the best cut-off of VAF to be used in clinical studies? Second, since systemic therapy for EOC includes also chemotherapy and often PARP inhibitors, do mtDNA mutations impact on response to these drugs? If mtDNA mutations associate with resistance to chemotherapy, as reported by some groups [57, 57, 57], which would be their net predictive effect?

In any case, given their potentially high translational value, we feel that our findings deserve validation in EOC and CRC patients treated with bevacizumab and other anti-angiogenic drugs.

## Conclusion

This study presents an analysis of mtDNA variants in OC patient-derived xenografts (PDXs) and demonstrates their multifaceted impact on cancer cells. For the first time, we reveal how experimental tumors carrying specific mtDNA variants respond to bevacizumab, a commonly used anti-angiogenic drug in cancer therapy. Our findings indicate that PDXs with homoplasmic pathogenic mtDNA variants exhibit improved survival under anti-VEGF treatment in mice, compared to PDXs with mtDNA wild-type or low heteroplasmy. These results hold potential for translation to OC patients, suggesting that mtDNA mutations could serve as predictive markers of positive response to bevacizumab and other anti-angiogenic therapies.

## Supplementary Information

Supplementary Material 1.

Supplementary Material 2.

## Data Availability

The data generated in this study are available within the article and its supplementary data files. RNA-seq data have been deposited in Gene Expression Omnibus (GEO), accession number GSE261674. mtDNA sequencing and WES data have been deposited in SRA, accession number PRJNA1122078.

## Abbreviations

mtDNA: Mitochondrial DNA
EOC: Epithelial ovarian cancer
PDX: Patient-derived xenografts
VEGF: Vascular endothelial growth factor
PFS: Progression free survival
OS: Overall survival
mRC: Mitochondrial respiratory chain
FFPE: Formalin-fixed paraffin-embedded
GDS: Glucose deprivation sensitive
GDR: Glucose deprivation resistant
WES: Whole exome sequencing
TEM: Transmission electron microscopy
DEGs: Differentially expressed genes
MCT4: Monocarboxylate transporter 4
POLG: DNA polymerase subunit gamma
ROS: Reactive oxygen species
CRC: Colon rectal cancer
