# Supplementary material for: Pathogenic mitochondrial DNA variants are associated with response to anti-VEGF therapy in ovarian cancer PDX models
Source: J Exp Clin Cancer Res. 2024 Dec 19;43:325. doi: 10.1186/s13046-024-03239-w (PMC11657443; doi:10.1186/s13046-024-03239-w)
Supplement: Supplementary file 2 — Supplementary Material 2. [file 13046_2024_3239_MOESM2_ESM.docx]

**Pathogenic mitochondrial DNA variants are associated with response to anti-VEGF therapy in ovarian cancer PDOVCA models**

Daniele Boso^1^*, Ilaria Piga^2^*, Chiara Trento^2^, Sonia Minuzzo^2^, Eleonora Angi^1^, Luisa Iommarini^3^, Elisabetta Lazzarini^1^, Leonardo Caporali^4^, Claudio Fiorini^4^, Luigi D'Angelo^3^, Monica De Luise^5,6^, Ivana Kurelac^5,6^, Matteo Fassan^7,8^, Anna Maria Porcelli^3,6,9^, Filippo Navaglia^10^, Ilaria Billato^11^, Giovanni Esposito^12^, Giuseppe Gasparre^5,6,13^, Chiara Romualdi^11^, Stefano Indraccolo^1,2^.

1. Basic and Translational Oncology Unit, Veneto Institute of Oncology IOV-IRCCS, Padova, Italy. 2. Department of Surgery, Oncology and Gastroenterology, University of Padova, Padova, Italy. 3. Department of Pharmacy and Biotechnology (FABIT), University of Bologna, Bologna, Italy. 4. IRCCS Istituto delle Scienze Neurologiche di Bologna, Bologna, Italy. 5. Department of Medical and Surgical Sciences (DIMEC), University of Bologna, Bologna, Italy. 6. Center for Applied Biomedical Research (CRBA), University of Bologna, Bologna, Italy. 7. Department of Medicine (DIMED), University of Padua, Padua, Italy  8. Veneto Institute of Oncology, IOV-IRCCS, Padua, Italy. 9. Interdepartmental Center for Industrial Research on Health Sciences and Technologies, University of Bologna, Bologna, Italy. 10. Laboratory Medicine, Department of Medicine-DIMED, University Hospital of Padova, Padova, Italy. 11. Department of Biology, University of Padova, Padova, Italy. 12. Immunology and Molecular Oncology Unit, Istituto Oncologico Veneto, IOV - IRCCS, Padova, Italy. 13. Centro Studi e Ricerca Sulle Neoplasie Ginecologiche (CSR), University of Bologna, Bologna, Italy.

**SUPPLEMENTARY MATERIALS AND METHODS**

**Ovarian cancer patient-derived xenografts (PDOVCAs) establishment and treatments**

Anti-VEGF treatment was administered as follows: for short term treatment experiments, PDOVCA 5 (controls n=3, treated n=8), PDOVCA 15 (controls n=4, treated n=5), PDOVCA 126 (controls n=4, treated n=7) and PDOVCA 62 (controls n=3, treated n=7) were intra-peritoneally treated with the anti-human VEGF monoclonal antibody bevacizumab (5 mg/kg, Roche, RRID:AB_2459640) two or three times when ascites were already present. For long term treatment experiments with PDOVCA 5, 62, 126, 49, 69, 15 and 17 (n=5 mice for the control group and n=5 as treated group) we started the intraperitoneal treatment twice per week with anti-VEGF at 20% of the estimated time to sacrifice until ascites formation was observed. Control mice received intraperitoneal injections of PBS. At sacrifice, ascites fluid containing cancer cells and blood samples were collected to perform further assays**.**

**Whole exome sequencing (WES)**

WES was performed on PDOVCA cells. Genomic DNA was extracted with QIAamp DNA Mini Kit (Qiagen, Hilden, Germany) following the manufacturer’s protocol and quantified with Qubit BR dsDNA Assay Kit (Invitrogen, Waltham, Massachusetts, USA). DNA samples (150 ng) were enzymatically fragmented by using SureSelect Enzymatic Fragmentation Kit and sequenced using the SureSelectXT Low Input Target Enrichment System Exome v7 (Agilent, Santa Clara, CA) on NextSeq 500 (Illumina, San Diego, CA) in paired-end mode (2x150 bp). Sequencing reads were then analyzed with the Agilent Technologies ALISSA Align & Call Software (version 3.3, Agilent Technologies, Santa Clara, CA). FASTQ reads were trimmed and aligned to the human genomic reference (hg19) with default parameters. Analysis was performed using the Alissa Interpret module (version 3.3, Agilent Technologies, Santa Clara, CA) on coding passing filters variants considering the cut-off value of 10% for the variant allele frequency (VAF), excluding both common polymorphisms, intronic variants and synonymous variants that do not alter splicing.

**DNA isolation from FFPE tissue biopsies**

DNA was extracted from cell pellets derived from PDOVCA ascites by using the QIAamp DNA mini extraction kit (Qiagen, Hilden, Germany, http://www.qiagen.com) according to the manufacturer’s instructions. DNA were extracted from formalin-fixed paraffin-embedded (FFPE) tissue biopsies of patients from which PDOVCAs were generated. DNA extraction from FFPE requires 5 slides of 10 μm of thickness to be used for DNA extraction and one slide with hematoxylin-eosin (HE) staining. HE stained slide was used to identify tumor areas with the help of an expert pathologist. In some cases, a macrodissection area was drawn to enrich the sample with tumor cells and exclude healthy or necrotic tissue. Slides were scraped using a scalpel and DNA was extracted using QIAamp DNA Micro Kit (Qiagen, Hilden, Germany), following manufacturer’s instructions. Elution of DNA was done in 30 μl nuclease-free water. For DNA quantification, Qubit assay was used, and the DNA concentration was measured by using the Qubit 4 Fluorometer (Invitrogen, Waltham, Massachusetts, USA).

**Histology, immunohistochemistry (IHC) and digital pathology**

For MT-CO1 staining, slides were digitally acquired at ×20 magnification using the Olympus VS200 Slide Scanner (EVIDENT Life Science). Evaluation of the IHC score was assessed through ImageJ, utilizing the "IHC Profiler" plugin (23). The method was modified as follows: after executing the plugin, the image underwent adjustments by creating a selection of stained cells using the threshold function of ImageJ. Subsequently, the automated IHC scoring macro was applied to facilitate the specific quantification of positive cells. At least 10 images per sample were analyzed. The results provided the percentage of cells with different expressions of proteins classified as 3+ (highly positive), 2+ (intermediate positive), 1+ (low positive), and 0 (negative). For statistics purposes, we unified 3+ and 2+ as positive cells and 1+ and 0 as negative. The digital quantification performed by the software was finally rewired and validated by an expert pathologist.

**SUPPLEMENTARY DATA**

| **Sample ID** | **mtDNA mutations** | **aa change** | **Heteroplasmy**  **VAF** | **Confirmed** |
| --- | --- | --- | --- | --- |
| **PDOVCA 1** | m.7763G>A/*MT-C02*  m.9412G>A/*MT-C03* | p.E60K  p.G69D | 17%  87% | N  N |
| **PDOVCA 6** | m.6691G>A/*MT-C01* | p.G263E | 100% | Y |
| **PDOVCA 9** | m.4686G>A/*MT-ND2*  m.5591G>A/*MT-TA* | p.A73T  - | 100%  100% | N  N |
| **PDOVCA 14** | m.13180G>A/*MT-ND5* | p.A282T | 22% | N |
| **PDOVCA 17** | m.11841T>C/*MT-ND4* | p.L361P | 2% | N |
| **PDOVCA 24** | m.8759T>C/ *MT-ATP6* | p.F78S | 17% | Y |
| **PDOVCA 36** | m.10197G>A/*MT-ND3* | p.A47T | 21% | Y |
| **PDOVCA 49** | m.1666T>C/*MT-TV*  m.6745G>A/*MT-C01* | p.G281D | 45%  53% | Y  Y |
| **PDOVCA 53** | m.6126A>G/*MT-CO1*  m.7030A>G/*MT-C01*  m.8529G>T/*MT- ATP8,ATP6* | p. 175V  p. H376R  p. WSSL, p.M1I | 72%  10%  15% | Y  N  N |
| **PDOVCA 54** | m.4142G>A/*MT-ND1* | p.R279Q | 36% | N |
| **PDOVCA 128** | m.4449G>A/*MT-TM* | - | 14% | N |

**Suppl. Table S1. mtDNA mutations comparison between PDOVCA and patients.** PDOVCA mutations were investigated in 11 matched DNA tumor samples, obtained from surgical specimens of the tumors at diagnosis. Mutations that were found in the corresponding patient DNA are indicated by letter Y (YES).

| **Sample**  **(PDOVCA)** | **Gene** | **Transcript** | **cDNA change** | **Protein change** | **Read Depth** | **VAF (%)** | **Pathogenicity prediction (VarSome)** |
| --- | --- | --- | --- | --- | --- | --- | --- |
| **1** | *TP53* | NM_001126114.2 | c.817C>T | p.(Arg273Cys) | 23 | 100 | P |
|  | *SUGCT* | NM_001193311.1 | c.273G>A | p.(Trp91*) | 93 | 16.1 | LP |
| **5** | *TP53* | NM_001126118.1 | c.880del | p.(Arg294Valfs*12) | 36 | 97.2 | P |
|  | *BRCA1* | NM_007294.3 | c.5266dup | p.(Gln1756Profs*74) | 53 | 90.6 | P |
|  | *SDHD* | NM_001276506.2 | c.364C>T | p.(Arg122*) | 67 | 32.8 | LP |
| **6** | *TP53* | NM_001126114.2 | c.415A>T | p.(Lys139*) | 59 | 100 | P |
|  | *BRCA2* | NM_000059.3 | c.5410_5411del | p.(Val1804Lysfs*2) | 85 | 98.8 | P |
|  | *GCK* | NM_001354800.1 | c.1107_1114del | p.(Ala370Glufs*99) | 85 | 24.7 | LP |
|  | *LFNG* | NM_001166355.1 | c.159_166dup | p.(Glu56Glyfs*144) | 63 | 28.6 | LP |
|  | *ATR* | NM_001184.4 | c.730del | p.(Ile244Leufs*4) | 161 | 52.8 | LP |
|  | *BEND3* | NM_001080450.2 | c.793del | p.(Asp265Thrfs*54) | 39 | 48.7 | LP |
|  | *GALT* | NM_000155.4 | c.658G>T | p.(Glu220*) | 111 | 53.2 | LP |
|  | *EEF2* | NM_001961.4 | c.490dup | p.(Leu164Profs*100) | 56 | 19.6 | LP |
| **9** | *TP53* | NM_001126114.2 | c.722C>T | p.(Ser241Phe) | 37 | 100 | P |
|  | *BRCA1* | NM_007294.3 | c.4389C>G | p.(Tyr1463*) | 147 | 100 | P |
| **14** | *PIK3CA* | NM_006218.2 | c.1031T>G | p.(Val344Gly) | 58 | 27.6 | LP |
|  | *ARID1A* | NM_006015.4 | c.4134_4153dup | p.(Glu1385Alafs*103) | 63 | 17.5 | LP |
|  | *STK11* | NM_000455.4 | c.164_165insA | p.(Glu57Glyfs*106) | 57 | 93 | LP |
| **15** | *TP53* | NM_001126114.2 | c.332T>A | p.(Leu111Gln) | 114 | 100 | P |
| **17** | *TP53* | NM_001126114.2 | c.659A>G | p.(Tyr220Cys) | 21 | 100 | P |
| **24** | *TP53* | NM_001126114.2 | c.764T>C | p.(Ile255Thr) | 115 | 100 | P |
| **41** | *BRCA2* | NM_000059.3 | c.3708dup | p.(Ala1237Serfs*6) | 86 | 96.5 | P |
|  | *TP53* | NM_001126114.2 | c.722C>T | p.(Ser241Phe) | 128 | 100 | P |
|  | *TRMT10C* | NM_017819.4 | c.584del | p.(Gly195Valfs*21) | 168 | 26.2 | LP |
| **49** | *TP53* | NM_001126114.2 | c.853G>A | p.(Glu285Lys) | 61 | 100 | P |
|  | *ALDOB* | NM_000035.4 | c.1005C>G | [p.(Asn335Lys)](https://iov.alissa.agilent.com/interpret/search_domains.html?type=variantPDot&query=NM_000035.4%3Ap.N335K&genomeBuildRoot=any&domainType=SOMATIC) | 53 | 52.8 | P |
|  | *MRNIP* | NM_016175.4 | c.486_498del | p.(Ser162Argfs*19) | 46 | 26.1 | LP |
| **52** | *TP53* | NM_001126114.2 | c.642_643del | p.(His214Glnfs*7) | 99 | 98.6 | P |
| **53** | *TP53* | NM_001126114.2 | c.395A>G | p.(Lys132Arg) | 131 | 99 | P |
| **54** | *TP53* | NM_001126114.2 | c.551_554del | p.(Asp184Alafs*62) | 81 | 98.8 | P |
|  | *KRAS* | NM_033360.4 | c.35G>A | p.(Gly12Asp) | 116 | 99.1 | P |
|  | *CASP8* | NM_001080125.2 | c.950_951insA | p.(Asp318Glyfs*17) | 45 | 40 | LP |
| **62** | *TP53* | NM_001126114.2 | c.643dup | p.(Ser215Lysfs*7) | 16 | 81.2 | P |
|  | *RET* | NM_020975.4 | c.1600del | p.(Leu534Trpfs*104) | 57 | 22.8 | LP |
| **126** | *TP53* | NM_001126114.2 | [c.578A>G](https://iov.alissa.agilent.com/interpret/search_domains.html?type=variantCDot&query=NM_001126114.2%3Ac.578A%3EG&genomeBuildRoot=any&domainType=SOMATIC) | p.(His193Arg) | 38 | 100 | P |
| **128** | *EBAG9* | NM_004215.5 | c.82A>T | p.(Arg28*) | 82 | 15.9 | LP |
| **145** | *TP53* | NM_001126114.2 | c.286del | p.(Ser96Leufs*27) | 156 | 94.2 | P |
|  | *MCCC1* | NM_020166.5 | c.1225C>T | p.(Arg409*) | 181 | 39.2 | P |
| **146** | *TP53* | NM_001126114.2 | c.533A>C | p.(His178Pro) | 88 | 100 | P |
|  | *BRCA2* | NM_000059.3 | c.6331_6332del | p.(Lys2111Glufs*17) | 137 | 29.9 | P |
|  | *BRCA2* | NM_000059.3 | c.6373del | p.(Thr2125Profs*12) | 113 | 72.6 | P |

**Suppl. Table S2. Whole exome sequencing (WES) analysis from DNA of 18 PDOVCA-derived cells.** Table describes the name of mutated gene, the gene isoform, the cDNA change and the relative protein change. Read depth, VAF (%) and the prediction of pathogenicity obtained with VarSome classifier are reported for each mutation in each PDOVCA (P pathogenic; LP likely pathogenic).


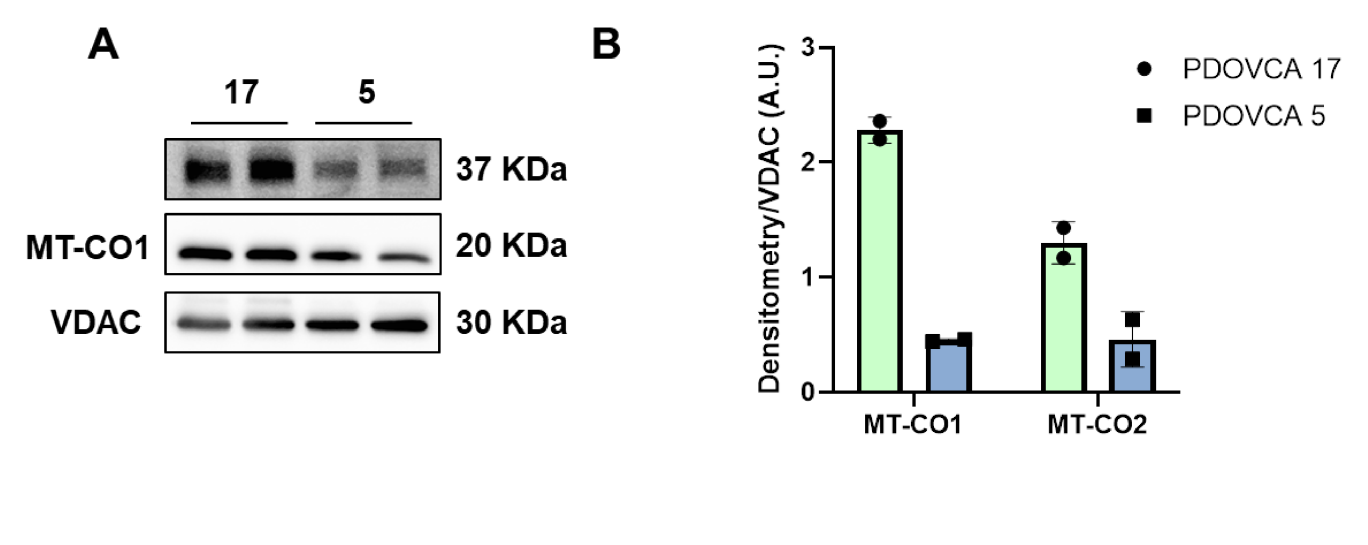


**Suppl. Figure S1. A.** Western blot of mitochondrial lysates obtained from PDOVCA5 (MUT) and PDOVCA17 (WT) separated by SDS-PAGE in which MT-CO1 and MT-CO2 were immune-detected.Voltage-dependent anion channels (VDAC) was used as loading control. **B.** Quantification of protein content in A. Data are represented as mean ± SD (n=2).


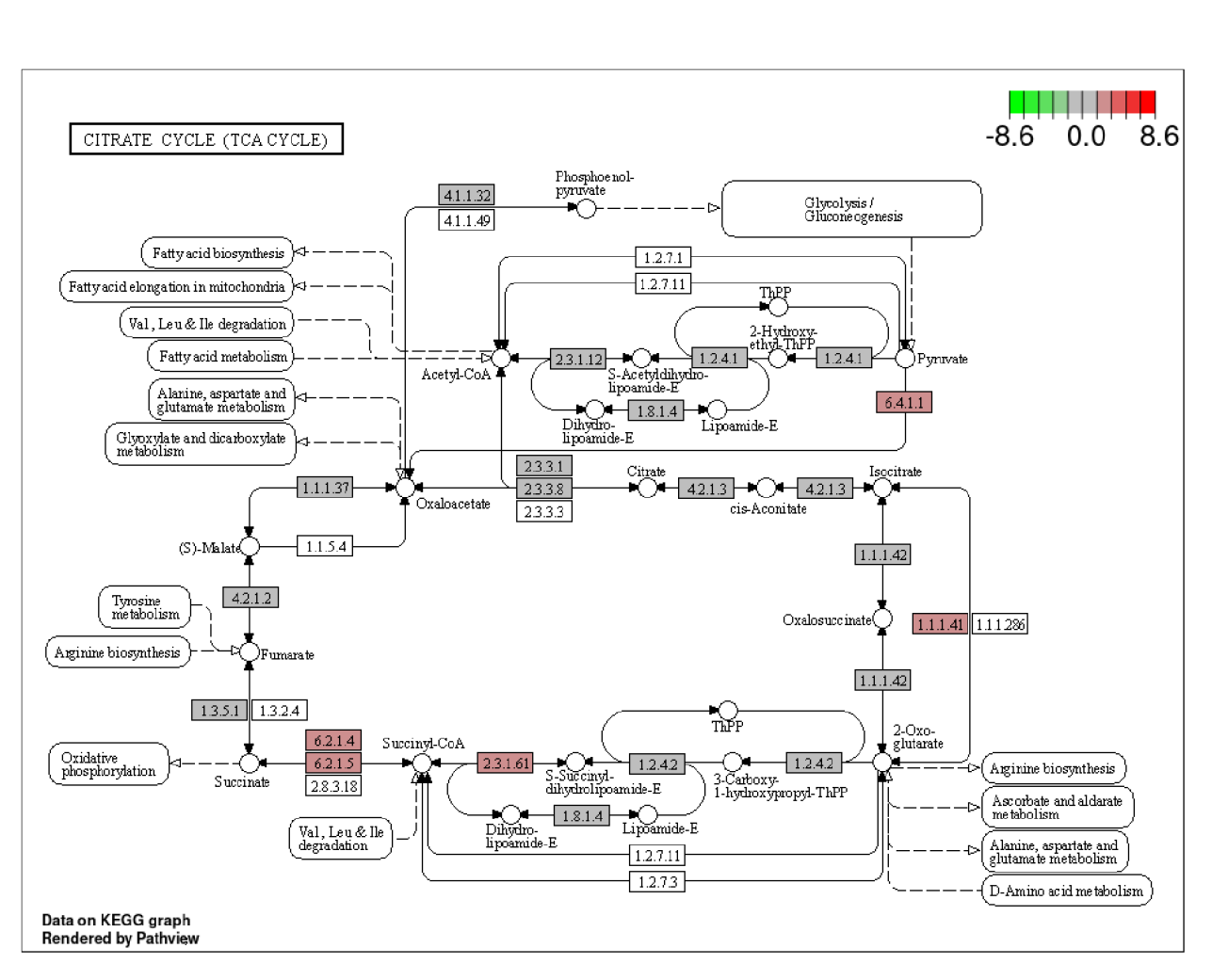


**Suppl. Figure S2. KEGG Citrate cycle (TCA cycle) pathway**. Up- and down- regulated genes in MUT vs WT PDOVCAs are highlighted in red and green, respectively.

**
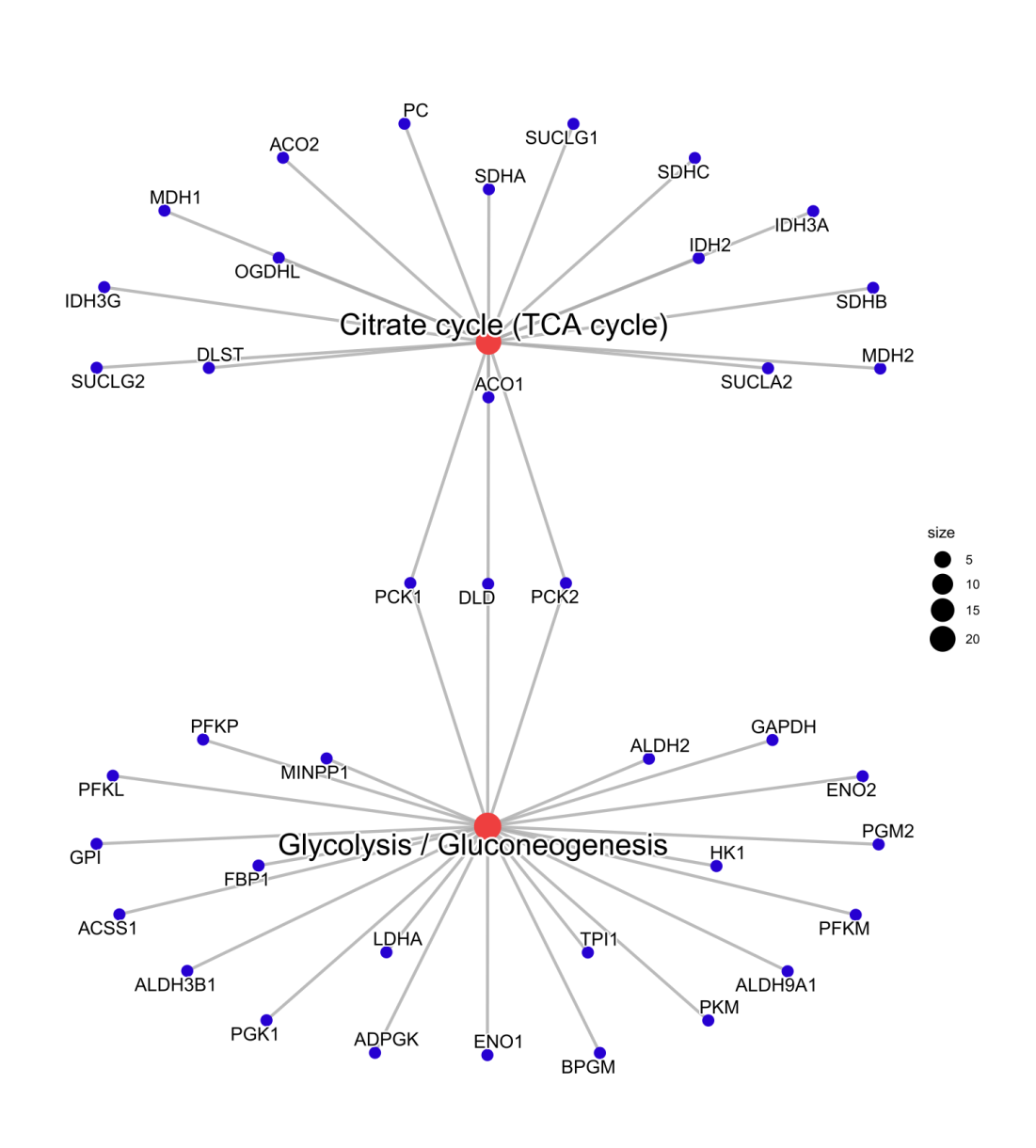
**

**Suppl. Figure S3. Gene-concept networks of enriched KEGG pathways citrate cycle (TCA cycle) and Glycolysis/ Gluconeogenesis**. Interconnections among core genes contributing to the positive enrichment of the two pathways between MUT versus WT PDOVCAs are visualized.


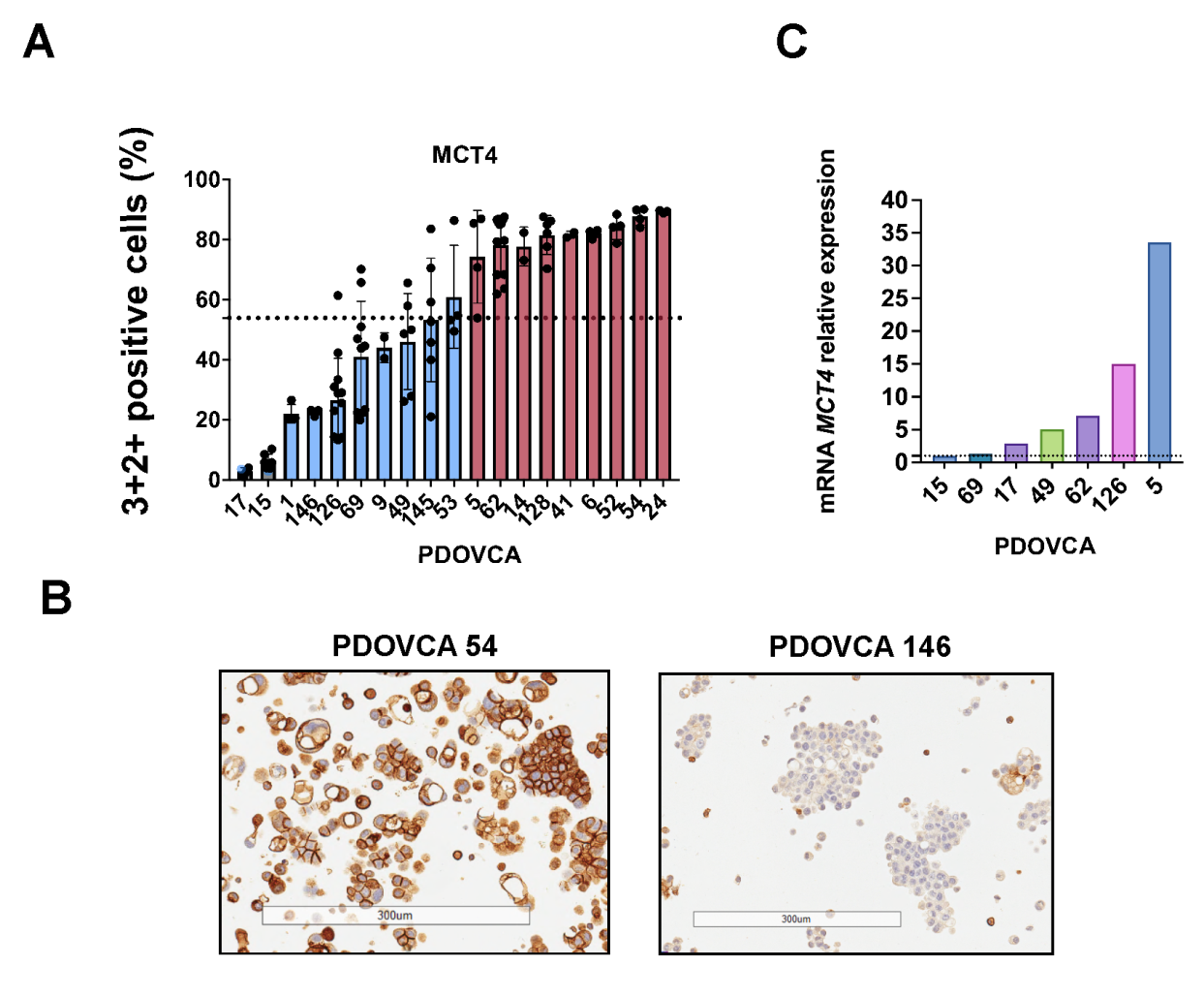


**Suppl. Figure S4. IHC of MCT4 monocarboxylate transporter 4 (MCT4) in PDOVCAs (n=19)**. **A.** Percentage of 3+ 2+ positive cells was calculated by digital pathology, as described in Materials and Methods. **B.** Representative images of IHC for PDOVCA54 (MCT4^high^) and PDOVCA146 (MCT4^low^). Scale bar=300 µM. **C.** Abundance of MCT4 transcripts in PDOVCAs (n=7). Relative expression was calculated by normalizing on *MCT4*^low^ PDOVCA15, as indicated by dotted line (n=1 for each PDOVCA).
